# Manufacturing Considerations for the Development of Lipid Nanoparticles Using Microfluidics

**DOI:** 10.3390/pharmaceutics12111095

**Published:** 2020-11-15

**Authors:** Carla B. Roces, Gustavo Lou, Nikita Jain, Suraj Abraham, Anitha Thomas, Gavin W. Halbert, Yvonne Perrie

**Affiliations:** 1 Strathclyde Institute of Pharmacy and Biomedical Sciences, University of Strathclyde, 161 Cathedral Street, Glasgow G4 0RE, UK; carla.roces-rodriguez@strath.ac.uk (C.B.R.); gustavo.lou-ramirez@strath.ac.uk (G.L.); g.w.halbert@strath.ac.uk (G.W.H.); 2 Precision NanoSystems Inc., #50 655 W Kent Ave N, Vancouver, BC V6P 6T7, Canada; njain@precision-nano.com (N.J.); sabraham@precision-nano.com (S.A.); athomas@precision-nano.com (A.T.)

**Keywords:** microfluidics, RNA, lipid nanoparticles, manufacture, critical process parameters, nucleic acid

## Abstract

In the recent of years, the use of lipid nanoparticles (LNPs) for RNA delivery has gained considerable attention, with a large number in the clinical pipeline as vaccine candidates or to treat a wide range of diseases. Microfluidics offers considerable advantages for their manufacture due to its scalability, reproducibility and fast preparation. Thus, in this study, we have evaluated operating and formulation parameters to be considered when developing LNPs. Among them, the flow rate ratio (FRR) and the total flow rate (TFR) have been shown to significantly influence the physicochemical characteristics of the produced particles. In particular, increasing the TFR or increasing the FRR decreased the particle size. The amino lipid choice (cationic—DOTAP and DDAB; ionisable—MC3), buffer choice (citrate buffer pH 6 or TRIS pH 7.4) and type of nucleic acid payload (PolyA, ssDNA or mRNA) have also been shown to have an impact on the characteristics of these LNPs. LNPs were shown to have a high (>90%) loading in all cases and were below 100 nm with a low polydispersity index (≤0.25). The results within this paper could be used as a guide for the development and scalable manufacture of LNP systems using microfluidics.

## 1. Introduction

RNA therapeutics is a promising approach to treat a wide range of medical conditions including cancer and infectious diseases [1]. However, these molecules are rapidly cleared or degraded upon administration, showing limited systemic potency in vivo [2]. In addition, their size and negative charge impede crossing the cell membrane [3]. Thus, specialised delivery systems are required for the efficient intracellular RNA delivery to the target site. Development of clinical therapeutic RNA is currently riding on a high, since the recent approvals of the first two siRNA drugs: patisiran (2018) and givosiran (2019) [4,5]. Patisiran is a lipid nanoparticle (LNP)-formulated siRNA used for the treatment of hereditary transthyretin-mediated amyloidosis. In contrast, givosiran is based on siRNA conjugated with a targeting moiety, which consists of three *N*-acetylgalactosamine (GalNAc) molecules. In addition to this, currently, a large number of vaccines are being developed against severe acute respiratory syndrome coronavirus 2 (SARS-CoV-2) using LNP technology for mRNA delivery (e.g., Moderna, CureVac, BioNTech) [1]. Nucleic acid-loaded LNP systems are complex structures (approximately 100 nm), typically composed of amino lipids (ionisable or cationic amino lipids) as the main component, phosphatidylcholine lipids, cholesterol and a polyethylene glycol-lipid conjugate (PEG-lipid). The development of ionisable cationic lipids has been a major breakthrough in the LNP field. These lipids are positively charged at an acidic pH and become neutrally charged at a physiological pH [6]. This quality grants them the advantage of electrostatically interacting with the negatively charged nucleic acid at a lower pH and allows the LNPs to deliver the cargo within the cytosol via an ApoE-mediated pathway, increasing LNP potency compared to the permanently charged cationic lipids. As a result, ionisable LNPs are not rapidly cleared by the reticuloendothelial system. Upon reaching the target cell, they become engulfed via endocytosis and become positively charged within the endosome (internal pH 5–6), fusing with the naturally occurring negatively charged endosomal lipids and therefore releasing the nucleic acid into the cytosol. For an optimal efficacy in the liver after intravenous administration, it has been demonstrated that ionisable lipids should have a pKa between 6.2 and 6.5 [7]. An example of this is the ionisable lipid Dlin-MC3-DMA, which has a pKa of 6.44 and is used within the approved patisiran formulation (Onpattro^®^, Alnylam Pharmaceuticals, Cambridge, MA, USA) [7,8]. PEG-lipids are also incorporated into the formulations to facilitate the production of nanometre-sized particles and to increase both particle stability, due to the steric interactions, and in vivo half-life. However, PEG-lipids may impair cellular uptake and therefore the use of “diffusible” PEG-lipids (which rapidly separate from the LNPs) is recommended for hepatic delivery. Thus, these lipid components have been shown to be critical and need to be at specific proportions within the LNP system [9].

Traditional methods for LNP manufacture have previously involved the production of a lipid film and subsequent hydration of the film with an aqueous buffer containing the nucleic acid to passively encapsulate the payload [10]. This commonly results in large (>100 nm) and heterogeneous particles with a low encapsulation yield, requiring the addition of a down-sizing technique such as extrusion or sonication. In addition to this, this method is difficult to scale-up and lacks reproducibility. Thus, the development of ethanol injection and microfluidic techniques during the early 2000s revolutionised LNP manufacturing [11,12]. Microfluidics is robust, scalable and very reproducible. It involves the mixture of lipids in an organic solvent with an aqueous phase containing the nucleic acid through a micromixer. The cationic lipid interacts with the negatively charged nucleic acid, resulting in high encapsulation efficiencies. LNPs of defined sizes can be accurately produced by controlling the microfluidic operating parameters such as flow rate ratio (FRR) or total flow rate (TFR). High production speeds (TFR) can be achieved using microfluidics, which results in a reduction in manufacturing time (up to 200 mL/min).

Here, we assessed the effects of formulation and microfluidic operating parameters during the production of LNPs, to provide practical information towards their development and manufacture. We evaluated the effects of the TFR and the FRR on LNP formulations containing either cationic (DOTAP-cLNPs or DDAB-cLNPs) or ionisable (MC3-iLNPs or GenVoy-ILM^TM^-iLNPs) lipids, which had previously been shown to be effective as LNP vaccine formulations [13]. In addition, formulation parameters such as lipid molar ratio composition, structural lipid, coating lipid and buffer choices were also evaluated. After formulation optimisation, PolyA, ssDNA and mRNA were encapsulated within the cLNPs and iLNPs, demonstrating the ability of microfluidic technology to accommodate a wide range of nucleic acids for LNP preparation.

## 2. Materials and Methods

### 2.1. Materials

Fully hydrogenated soy phosphatidylcholine (HSPC), 1,2-distearoyl-sn-glycero-3-phosphocholine (DSPC) and 1,2-distearoyl-sn-glycero-3-phosphoethanolamine-*N*-[methoxy (polyethylene glycol)-2000] (DSPE-PEG2000) were obtained from Lipoid (Ludwigshafen, Germany). 1,2-dioleoyl-3-trimethylammoniumpropane (chloride salt) (DOTAP), dimethyldioctadecylammonium (Bromide Salt) (DDAB) and 1,2-dimyristoyl-rac-glycero-3-methoxypolyethylene glycol-2000 (DMG-PEG2000) were purchased from Avanti Polar Lipids (Alabaster, AL, USA). Cholesterol (Chol), citric acid, sodium citrate tribasic dehydrate, polyadenylic acid (PolyA) and single stranded deoxyribonucleic acid (ssDNA) from salmon testes were acquired from Sigma-Aldrich (St. Louis, MO, USA). Phosphate-buffered saline tablets (PBS pH 7.4) were acquired from Oxoid Ltd. (Basingstoke, UK). Tris (hydroxymethyl) aminomethane (TRIS-base) and ethanol (EtOH) were obtained from Fisher Scientific, Loughborough, UK. The ionisable lipid heptatriaconta-6, 9, 28, 31-tetraen-19-yl 4-(dimethylamino) butanoate (Dlin-MC3-DMA or MC3) was purchased from Biorbyt Limited (Cambridge, UK). GenVoy-ILM^TM^ is a commercially available proprietary lipid mix composition comprising DSPC:cholesterol:ionisable lipid:stabilizer at 10:37.5:50:2.5 mol% for encapsulating nucleic acids, manufactured by Precision NanoSystems Inc. Messenger RNA (CleanCap^®^ mRNA Fluc, L-7602) from TriLink BioTechnologies (San Diego, CA, USA) was gifted by Precision NanoSystems Inc. (Vancouver, BC, Canada). All solvents and other chemicals were of analytical grade, and milliQ-water was provided by an in-house system.

### 2.2. Microfluidics

**Figure 1:**
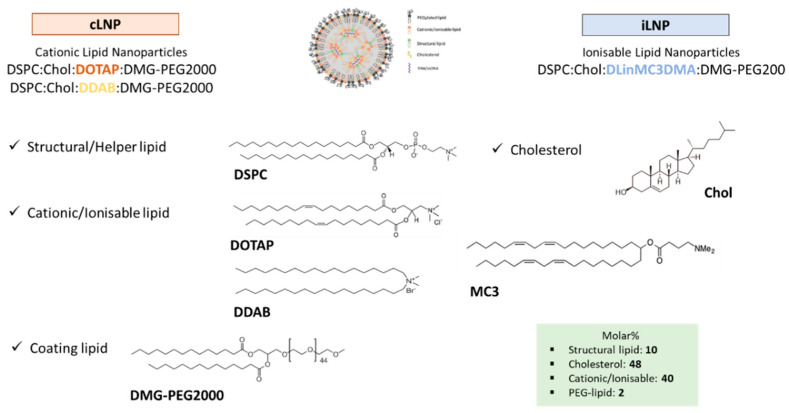
Lipid nanoparticles selected for optimisation. Cationic lipid nanoparticles (cLNPs) are based on either the cationic lipid DOTAP or DDAB, whereas ionisable lipid nanoparticles (iLNPs) are based on the ionisable lipid DLin-MC3-DMA. All described formulations contain DSPC and cholesterol as structural lipids and DMG-PEG2000 as the coating lipid. Unless otherwise stated, the lipid molar ratio used was 10:48:40:2 for DSPC:cholesterol:cationic/ionisable:PEG-lipid. The molecular structures of each component are shown in the figure along with the schematic representation of the structure of a lipid nanoparticle loaded with nucleic acids.

Lipid nanoparticles (LNPs) were prepared using the NanoAssemblr^®^ Benchtop from Precision NanoSystems Inc. (Vancouver, BC, Canada) which uses a Y-shape staggered herringbone micromixer [14]. Cationic lipid nanoparticles (cLNPs) were composed of DSPC:Chol:DOTAP:DMG-PEG2000 or DSPC:Chol:DDAB:DMG-PEG2000, whereas ionisable lipid nanoparticles (iLNPs) were composed of DSPC:Chol:MC3:DMG-PEG2000. Individual lipid stocks were prepared in ethanol. Unless otherwise stated, lipid mixtures were dissolved in ethanol at 10:48:40:2 molar ratio for Structural:Chol:cationic/ionisable:PEG-lipid respectively (based on research carried out by Geall et al. [15]). The final lipid concentration after microfluidic production was 2 mg/mL in all cases. Citrate buffer pH 6 100 mM was used as aqueous phase. The effect of the production speed (TFR; 5-20 mL/min) and the aqueous-to-organic ratio (FRR; 1:1 to 5:1) was evaluated. Effect of the condensing lipid (DDAB, DOTAP or MC3), coating lipid (DMG-PEG2000 vs. DSPE-PEG2000) and structural lipid choice (HSPC vs. DSPC) was assessed. Besides, the effect of the lipid molar ratio and the choice of aqueous buffer (citrate buffer pH 6 100 mM vs. TRIS buffer pH 7.4 10 mM) was studied. For the preparation of cLNPs or iLNPs encapsulating either PolyA, ssDNA or mRNA, these were added into the aqueous phase (citrate buffer pH 6, 100 mM) at the nitrogen-to-phosphate ratio (N/P; nitrogen from the cationic/ionisable lipid and phosphate from the nucleic acid) of 8. Initial and final waste volumes were set at 0.25 and 0.05 mL respectively. To facilitate the explanation of the experiments we will refer to the LNPs produced with DOTAP or DDAB as cationic lipid nanoparticles (cLNPs) and the LNPs containing the ionisable lipid MC3 as ionisable lipid nanoparticles (iLNPs) (Figure 1). Formulations were dialysed (MWCO 12,000–14,000 Da, Sigma-Aldrich, St. Louis, MO, USA) for 1 h under magnetic stirring against 200 mL PBS or TRIS buffer for iLNPs (MC3) and cLNPs (DDAB or DOTAP) respectively. This process is sufficient to reduce residual solvent levels to below 5000 ppm (results not shown). Whilst generally PBS is the default choice of buffer, TRIS buffer was adopted for cLNPs, as cationic lipid-based systems are sensitive to high salt buffers such as PBS and are known to precipitate.

### 2.3. Scalable Production of Lipid Nanoparticles Using a Toroidal Micromixer (TrM)

GenVoy-ILM™, a proprietary ionisable lipid mix formulation loaded with PolyA (N/P 6), was produced using different microfluidic mixers: a staggered herringbone (SHM) and a toroidal mixer (TrM) (NanoAssemblr Classic and NxGen™ respectively; Precision NanoSystems Inc., Vancouver, BC, Canada). GMP microfluidic production of LNPs was achieved using the NanoAssemblr GMP system and a TrM (NxGen 500) cartridge (Precision NanoSystems Inc. (Vancouver, BC, Canada), which uses the same toroidal mixer design with inline dilution and with custom HPLC pumps. Production speeds from 12 mL/min up to 200 mL/min were tested. The produced GenVoy-ILM™-based Poly (A) iLNP nanoparticles were diluted to an ethanol concentration below 1%. An aliquot of the GenVoy-ILM™ iLNP formulations were further ultra-centrifuged at 3000 RPM using 10 kDa MWCO Amicon Ultrafiltration units (Sigma-Aldrich, St. Louis, MO, USA) for the removal of solvent.

### 2.4. LNP Characterisation: Particle Size, Polydispersity and Zeta Potential

Particle size (Z-average diameter), polydispersity index (PDI) and zeta potential (ZP) were measured by dynamic light scattering (DLS) using a Zetasizer Nano ZS (Malvern Panalytical Ltd., Worcestershire, UK) equipped with a 633 nm laser and a detection angle of 173°. LNPs were diluted in filtered (0.22 µm) ultrapure water down to 0.2 mg/mL LNP concentration. 1000 µL of diluted particles were added into a 4-mL cuvette. The same dilution was used for zeta potential measurements. The dispersant (water) refractive index (RI) and viscosity values were 1.330 and 0.8872 cP respectively, whereas the material absorbance and RI were 0.01 and 1.49 respectively. Zetasizer Software v.7.11 (Malvern Panalytical Ltd., Worcestershire, UK) was used for the acquisition of data. All measurements were undertaken in triplicate with the attenuation value between 7 and 8.

### 2.5. TNS Fluorescence Titration Assay

The surface pKa of the ionisable lipid contained in the GenVoy-ILM™ formulation was studied alongside DSPC:Chol:DOTAP:DMG-PEG2000 cLNPs (10:38.5:50:1.5 mol%). Amino lipid pKa values were determined for each LNP system by measuring the fluorescence of 2-(*p*-toluidino)-6-napthalene sulfonic acid (TNS) during titration with buffer solutions with pH from 3.5 to 9 with 0.5 pH unit increments (10 mM HEPES, 130 mM NaCl, 10 mM NH_4_Oac, 10 mM MES). Formulation were diluted to 0.78 mM with milliQ-water and 6.4 µL was added to a black 96-well plate with 233.6 µL of pH buffer and 10 µL of 25 µM TNS solution. Fluorescence was quantified at λem = 445 nm, λex = 321 nm. Sigmoidal best-fit analyses were applied to the titration curves, and pKa, defined as pH at half-maximal fluorescence intensity, was observed.

### 2.6. Quantification of Nucleic Acid Loading: Poly(A), ssDNA and mRNA

Nucleic acid (PolyA, ssDNA and mRNA) encapsulation efficiency (EE%) was measured using Quant-iT™ RiboGreen™ RNA Assay Kit (Invitrogen™, Thermo Fisher Scientific, Waltham, MA, USA). Briefly, 100 μL of the diluted fluorescent dye was added to 100 μL of diluted liposomes in the presence or absence of 1% (*w*/*v*) Triton-X and incubated in absence of light for 5 min. Nucleic acids were quantified by measuring fluorescence (λem = 520 nm, λex = 480 nm) using a fluorimeter (Polarstar Omega, BMG Labtech). A linear calibration curve up to 1500 ng/mL was obtained (R^2^ ≥ 0.998) for each of the nucleic acid tested. Calculated limit of detection (LOD) and limit of quantification (LOQ) were 73 and 221 ng/mL, 61 and 184 ng/mL and 90 and 272 ng/mL for PolyA, ssDNA and mRNA respectively and all analyses were performed between LOQ and 1500 ng/mL.

### 2.7. Cryogenic Transmission Electron Microscopy (Cryo-TEM)

Cryo-TEM images of the blank and mRNA-cLNPs for microscopy were prepared by placing 5 μL of LNPs onto a 400-mesh lacey carbon-coated grid using single-sided blotting for 2 s, then immediately immersing the sample grid into nitrogen-cooled ethane (100% ethane). LNP morphology was then observed using the Jeol Jem F-200 microscope (Joel, Tokyo, Japan) at liquid nitrogen temperature and 200 kV.

Cryo-TEM images of the blank and GenVoy-ILM™ encapsulated mRNA iLNPs were performed as per Belliveau et al. [16] with slight modifications. The mRNA LNPs used for CryoTEM imaging were downstream processed and concentrated to the desired RNA concentration using 10 kDa MWCO Amicon Ultra filter units. Briefly, LNPs were approximately concentrated to more than the target RNA concentration using Amicon Ultra Filtration. The concentration was assessed by the modified Quant-iT™ RiboGreen™ RNA Assay and adjusted using 0.1 × PBS. Alternatively, if the concentration was found to be below the target concentration, the ultrafiltration using Amicon Ultra filters was continued until the final target concentration is achieved. The concentrated samples were then prepared by applying 3 µL of LNPs to a glow discharged Lacey Carbon 300 mesh copper TEM grid. Excess liquid was removed by blotting with a Vitrobot™ Mark VI robots (Thermo Fisher Scientific, Waltham, MA, USA) and then plunge-freezing the sample in liquid ethane to rapidly freeze the vesicles in a thin film of amorphous ice. Samples were cryogenically imaged using a 300 kV FEI Titan Krios TEM with a high-resolution Falcon III Electron detector.

### 2.8. Statistical Analysis

Results are represented as mean ± SD of at least n = 3 independent batches. ANOVA tests were used to assess statistical significance, with a Tukey’s post ad-hoc test (*p*-value of less than 0.05).

## 3. Results

### 3.1. Method Optimisation for Lipid Nanoparticles (LNPs)

#### 3.1.1. Effect of Microfluidic Operating Parameters: Total Flow Rate and Flow Rate Ratio

**Figure 2:**
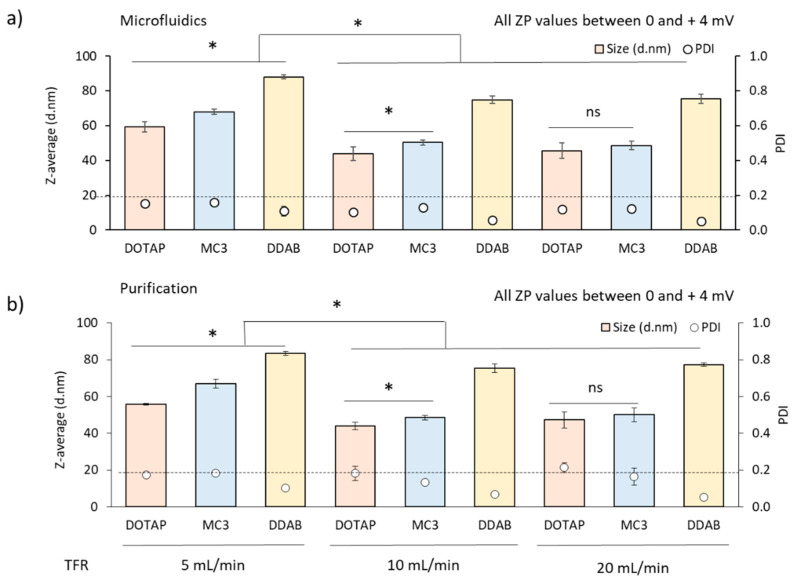
Effect of the total flow rate (TFR) on the physicochemical attributes of DOTAP (pink), MC3 (blue) and DDAB-based (yellow) lipid nanoparticles produced by microfluidics at 3:1 FRR using citrate buffer pH 6 100 mM as an aqueous buffer. cLNPs were purified via dialysis using TRIS buffer pH 7.4 10 mM whereas iLNPs were dialysed against PBS pH 7.3 10 mM. Particle size (bars) and PDI (open circles) of the LNPs produced using microfluidics before (**a**) and after (**b**) purification via dialysis. Results represent the mean of at least 3 independent batches and the standard deviation is plotted as error bars. ZP (zeta potential). Significant differences are shown as * *p* < 0.05and ‘ns’, when no statistical significance observed.

Initially, cationic lipid nanoparticles (cLNPs) and ionisable lipid nanoparticles (iLNPs) were produced by mixing lipids in ethanol with citrate buffer (pH 6 100 mM) using a staggered herringbone micromixer, followed by dialysis in TRIS buffer (for cLNPs) or PBS (for iLNPs) at pH 7.3–7.4 to remove the organic solvent and raise the pH to physiological conditions. All three LNP systems contained four lipid components namely DSPC, cholesterol, DMG-PEG2000 and a cationic lipid (either DDAB, DOTAP or MC3) at 10:40:48:2 molar percentage and 2 mg/mL final lipid concentration after microfluidics production. Important parameters to optimise using microfluidics are the TFR and the FRR. We started by evaluating the effect of the TFR at a fixed FRR (3:1). Figure 2 shows the particle size, PDI and zeta potential of DDAB and DOTAP cLNPs and MC3 iLNPs produced at varying speeds (from 5 to 20 mL/min) before (Figure 2a) and after purification via dialysis (Figure 2b). In general, particle size decreased with increasing TFR from 5 to 10–20 mL/min. The size of the LNPs stabilised at TFR 10–20 mL/min, with no significant differences observed across these production speeds. DOTAP-cLNPs showed the smallest particle size at all TFRs tested, followed by iLNPs and DDAB-cLNPs. The size of DOTAP-cLNPs ranged from 60 to 45 nm, MC3-iLNPs from 68 to 50 nm and DDAB-cLNPs from 88 to 75 nm for 5 to 20 mL/min, respectively (Figure 2). All LNPs were homogeneous in nature (PDI ≤ 0.2), with DDAB-cLNPs showing the most homogeneous particle sizes (PDI ≤ 0.1) (Figure 2). Purification of the LNPs by dialysis did not impact upon their particle size. Zeta potential values were neutral for all formulations across the speeds tested (Figure 2). Regardless of the choice of cationic lipid, the effect of this microfluidic parameter was very similar, with low production speed forming larger particles and higher speeds decreasing the particle size.

**Figure 3:**
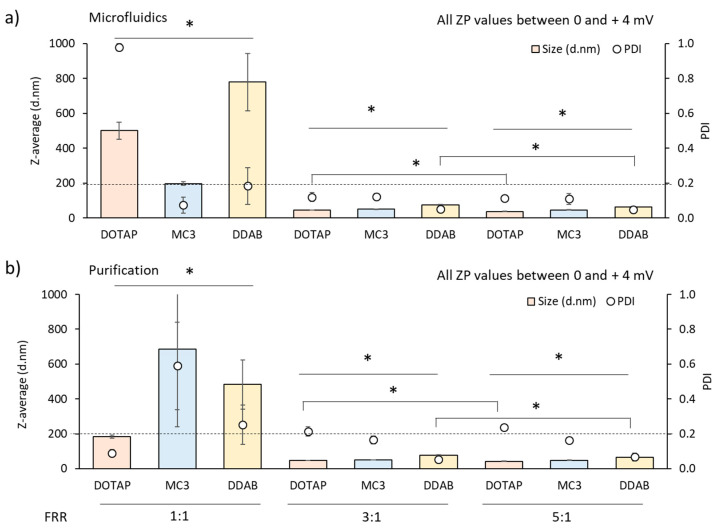
Effect of the flow rate ratio (FRR) on the production of lipid nanoparticles based either on DOTAP (pink), MC3 (blue) or DDAB (yellow) at 20 mL/min TFR using citrate buffer pH 6 100 mM as production buffer. cLNPs were purified via dialysis using TRIS buffer pH 7.4 10 mM whereas iLNPs were dialysed against PBS pH 7.3 10 mM. Particle size (bars) and PDI (open circles) of the LNPs produced using microfluidics (**a**) before and (**b**) after purification via dialysis. Results represent the mean of at least 3 independent batches and the standard deviation is plotted as error bars. ZP (zeta potential). Significant differences are shown as * *p* < 0.05.

After evaluation of the TFR, the next step was to assess the effect of the ratio between the aqueous (citrate buffer pH 6, 100 mM) and the organic (lipids in ethanol) phases (FRR, Figure 3). In order to accelerate the production of these LNP systems, the highest flow rate (20 mL/min) was selected for further experimentation. We varied the FRR from 1:1 to 5:1, and all LNP formulations showed a similar trend. As the FRR was increased, particle size decreased (Figure 3). LNPs produced at 1:1 FRR resulted in the largest particle sizes. DOTAP-cLNPs had a hydrodynamic size of 500 nm and a high heterodispersity (PDI > 0.8). DDAB-cLNPs were approximately 800 nm in size with a PDI of 0.2, while MC3-iLNPs were 200 nm in size, showing PDI values below 0.2 (Figure 3a). However, the physicochemical characteristics of the LNPs produced at 1:1 FRR changed after dialysis (Figure 3b). In the case of cLNPs, both DOTAP and DDAB formulations decreased in particle size down to 180 and 480 nm respectively, while MC3-iLNPs significantly (*p* < 0.05) increased in size (up to approximately 700 nm) (Figure 3). In the case of the PDI of the formulations before and after purification, DOTAP significantly (*p* < 0.05) decreased to 0.1, MC3 increased to 0.6 while DDAB PDI remained unchanged. When comparing 3:1 and 5:1 FRR, cLNPs significantly (*p* < 0.05) decreased in size from 46 to 37 nm and from 75 to 65 nm for DOTAP and DDAB-cLNPs respectively. MC3-iLNPs resulted in sizes of approximately 50 nm at both FRR. All three LNPs showed homogeneous size distributions (PDI ≤ 0.25), with DDAB-cLNPs showing significantly (*p* < 0.05) higher homogeneity (PDI ≤ 0.1) (Figure 3). The zeta potential remained comparable across all three FRR tested.

#### 3.1.2. Effect of the Lipid Molar Ratio

**Figure 4:**
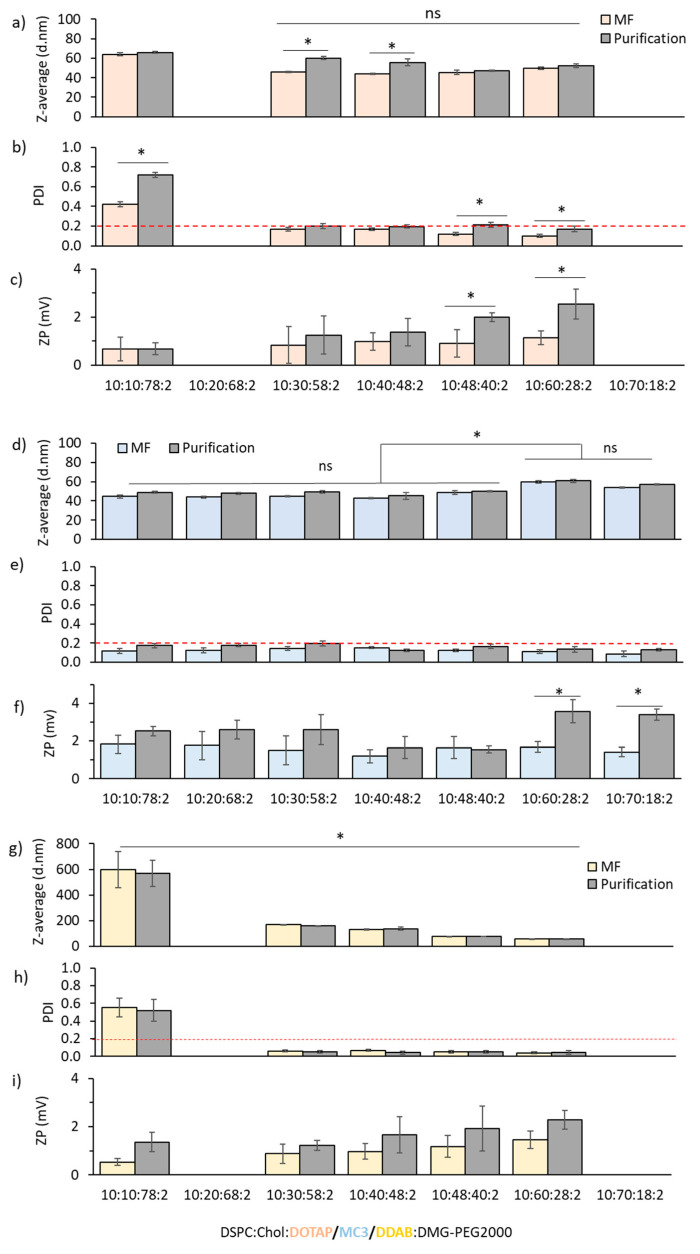
Effect of the molar ratio composition on DOTAP-cLNPs, MC3-iLNPs and DDAB-cLNPs. The molar ratio of DSPC and DMG-PEG2000 was kept constant at 10 and 2 mol% respectively; however, the ratio of cholesterol and the cationic lipid DDAB was varied from 10–60 and from 78–28 mol% respectively. (**a**,**d**,**g**) Particle size, (**b**,**e**,**h**) PDI and (**c**,**f**,**i**) zeta potential (ZP) of the DOTAP-cLNPs (pink), MC3-iLNPs (blue) and DDAB-cLNPs (yellow) after microfluidics and after purification via dialysis (grey). Results represent the mean of at least 3 independent batches and the standard deviation is plotted as error bars. Significant differences are shown as * *p* < 0.05 and ‘ns’, when no statistical significance observed.

**Figure 5:**
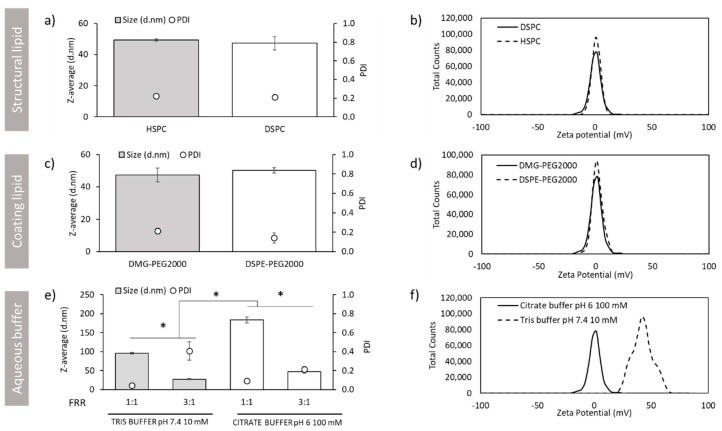
Effect of the structural lipid, the coating lipid and the aqueous buffer choice in the production of cLNPs based on DOTAP after purification via dialysis. (**a**) Particle size (bars) and PDI (open circles) and (**b**) zeta potential intensities of the cLNPs produced using HSPC (grey) or DSPC (white) as structural lipid. (**c**) Particle size (bars) and PDI (open circles) and (**d**) zeta potential intensities of the cLNPs produced using DMG-PEG2000 (grey bar) or DSPE-PEG2000 (white bar) LNPs. (**e**) Particle size (bars) and PDI (open circles) and (**f**) zeta potential intensities of the cLNPs produced using TRIS buffer pH 7.4 10 mM (grey) or citrate buffer pH 6 100 mM as production buffer. Unless otherwise stated, all the formulations were prepared at 20 mL/min TFR, 3:1 FRR and using citrate buffer as production buffer (pH 6, 100 mM). Results represent the mean of at least 3 independent batches and the standard deviation is plotted as error bars. Significant differences are shown as * *p* < 0.05.

**Figure 6:**
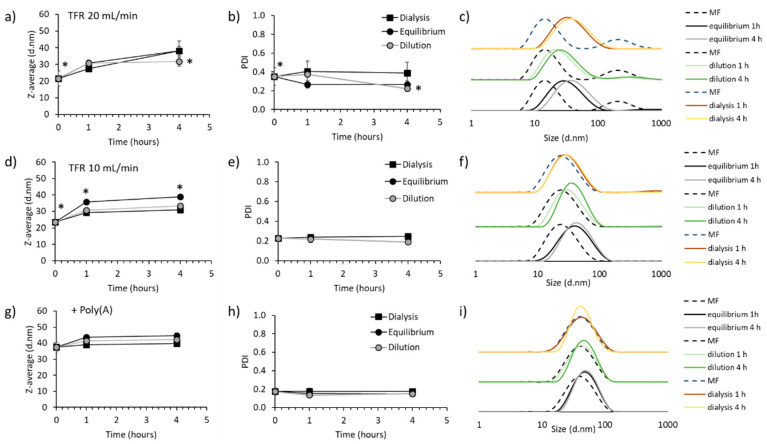
Stability study on the production of DOTAP-cLNPs produced at 3:1 FRR using TRIS pH 7.4 10 mM as the buffer choice. cLNPs were produced using microfluidics (MF) followed by either dilution in citrate buffer (5-fold), dialysis against TRIS buffer or equilibrated at room temperature (no solvent removal). DLS measurements were taken at times 0, 1 and 4 h. (**a**) Particle size, (**b**) PDI, and (**c**) intensity plots of empty DOTAP-cLNPs produced at 20 mL/min TFR. (**d**) Particle size, (**e**) PDI, and (**f**) intensity plots of empty DOTAP-cLNPs produced at 10 mL/min TFR. (**g**) Particle size, (**h**) PDI, and (**i**) intensity plots of empty DOTAP-cLNPs loading PolyA at N/P 8 and produced at 20 mL/min TFR. Results represent the mean of at least 3 independent batches and the standard deviation is plotted as error bars. Significant differences are shown as * *p* < 0.05.

Next, we sought how the composition (i.e., lipid molar ratio) affected the particle size, PDI and surface charge of these LNP formulations (Figure 4, Figure 5 and Figure 6). The PEG-lipid and structural lipid DSPC were fixed at 2 and 10 mol% respectively. However, the ratio between cholesterol and the cationic lipid was varied. The operating microfluidic parameters applied for the assessment of the lipid molar ratio effect were 3:1 FRR and 20 mL/min TFR (based upon the previous results). Figure 4 shows the effect of the variation of both cholesterol and cationic lipid molar ratio for the DOTAP formulation. At low cholesterol content (10 mol%), DOTAP-cLNPs displayed particle sizes of 65 nm and a high PDI (≥0.4), which further increased after purification (PDI ≥ 0.7) (Figure 4a,b). Increasing cholesterol molar ratio, and consequently decreasing the ratio of cationic lipid, resulted in a decrease in particle size and PDI. Particle sizes increased after purification until approximately 50% cholesterol was included into the formulation, where DOTAP-cLNPs showed comparable particles sizes before and after dialysis (Figure 4a). Zeta potential values ranged from 0 to +3 mV (Figure 4c).

The effect of the molar ratio of the formulation components on the physicochemical characteristics of MC3-iLNPs was then evaluated (Figure 4d–f). Unlike DOTAP-cLNPs, varying the cholesterol content from 10 to 48 mol% (and therefore reducing MC3 lipid from 78 to 40 mol%), did not affect the particle size nor the PDI of the particles produced after microfluidics (Figure 4d,e). These formulations ranged from 45 to 50 nm and showed high homogeneous size populations (PDI ≤ 0.2). Only when 60 or 70 mol% cholesterol was chosen, the size of these particles significantly (*p* < 0.05) increased up to 70 nm. Opposite to DOTAP-cLNPs, purification of these particles did not affect the characteristics at any of the mol% tested (Figure 4d,e). Zeta potential values were neutral for all the formulations (Figure 4f).

Figure 4g–i shows the effect of the lipid ratio content on DDAB-cLNPs. In general, increasing the cholesterol content from 10 to 60 mol% decreased the particle size in a steady manner from 600 nm down to 56 nm. Particles containing a low cholesterol molar ratio (10 mol%) were the least stable, showing large sizes and high PDI values (≈0.6) (Figure 4g,h). However, all other compositions tested resulted in highly monodisperse size distributions, with PDI values below 0.1. DDAB-cLNPs were purified via dialysis without modifying the physicochemical characteristics (Figure 4g,h). Zeta potential values were neutral for all the formulations (Figure 4i).

#### 3.1.3. Effect of the Choice of Structural Lipid, Coating Lipid and Buffer on the Physicochemical Characteristics of DOTAP-cLNPs

Previously, we have shown the physicochemical characteristics of LNPs produced using microfluidics with DSPC as a structural lipid and DMG-PEG2000 as the coating lipid. Figure 5a and b show the effect of replacing DSPC for HSPC on the particle size, PDI and zeta potential of DOTAP-cLNPs after purification. Microfluidic operating parameters were kept constant at 3:1 FRR and 20 mL/min TFR. No significant differences were observed among these lipids, with particle sizes of approximately 50 nm, PDI values of 0.2 and neutral zeta potentials for both DSPC and HSPC-containing formulations. The same results were observed when the PEG-lipid was substituted for a longer hydrocarbon chain lipid (Figure 5c,d). Replacement of DMG-PEG2000 for DSPE-PEG2000 resulted in DOTAP-cLNPs with comparable physicochemical characteristics (Figure 5c,d).

Another important parameter during LNP manufacture is the ionic buffer strength and pH. Citrate buffer at low pH is often used in the preparation of RNA-LNPs containing ionisable/cationic lipids to decrease base hydrolysis of RNA. However, we sought to examine the effect that TRIS 10 mM pH 7.4 had on the production of DOTAP-cLNP (Figure 5e,f). Additionally, we evaluated the 1:1 and 3:1 FRR. At first glance, we see that cLNPs produced using TRIS buffer are smaller (95 and 25 nm for 1:1 and 3:1 FRR respectively) compared to cLNPs produced using citrate buffer (180 and 50 nm for 1:1 and 3:1 FRR respectively) (Figure 5e). Furthermore, DOTAP-cLNPs at 3:1 FRR using TRIS were significantly (*p* < 0.05) more heterodisperse (PDI approx. 0.4) compared to those formulated in citrate buffer. In contrast, the rest of formulations tested showed PDI values below 0.2 (Figure 5e) regardless of the buffer choice. The effect of the production buffer choice was shown to impact upon the zeta potential as well. DOTAP-cLNPs produced using TRIS resulted in cationic zeta potential values between +20 mV and +40 mV, while particles produced using citrate buffer were neutral (Figure 5f).

Given that the effect of buffer selection during LNP manufacture had a notable impact upon the final LNP characteristics, we wanted to further explore why TRIS buffer at 3:1 FRR resulted in a highly heterogeneous particle distribution (Figure 6). Therefore, a stability study was carried out where these particles were produced using microfluidics (MF, t_0h_) followed by either: (1) dialysis in TRIS for 1 h, (2) 5-fold dilution in TRIS (to reduce solvent content down to 5% *v*/*v*) or (3) left in 25% solvent (labelled equilibrium). After each procedure, DLS measurements were performed after 1 h and 4 h. Initially, 20 mL/min TFR was tested (Figure 6a–c). DOTAP-cLNPs showed small particle sizes (≈25 nm) and high PDI (≈0.4) at t_0h_. After both dialysis and equilibrium procedures, DOTAP-cLNPs increased up to 30 and 40 nm after 1 and 4 h respectively (Figure 6a). However, after dilution in TRIS buffer, the particles remained stable at 30 nm for 4 h, while the PDI decreased down to 0.2 at t_4h_. Equilibrium also improved the PDI, however the particle size continued to increase over time (Figure 6b). Figure 6c shows the DLS intensity plots of the same conditions. Next, we decreased the TFR down to 10 mL/min (Figure 6d–f) where, likewise, the particle size after microfluidics was approximately 25 nm. However, in this case, the PDI following microfluidics (t_0h_) was improved (PDI ≤ 0.25). After dilution and dialysis, DOTAP-cLNPs increased in size up to 30 nm (Figure 6d). When particles remained in solvent under equilibrium, a significantly (*p* < 0.05) higher increase (up to 40 nm) was observed. All the particles produced at 10 mL/min showed narrow and unimodal size distributions, as seen on Figure 6e and f. Finally, these stability conditions were analysed when nucleic acid (PolyA N/P 8) was encapsulated within the formulation (DOTAP-cLNPs) during manufacture (t_0h_) using a TFR of 20 mL/min. The addition of the nucleic acid is shown to act as a stabiliser, where particle sizes between 40 and 45 nm and monodisperse size distributions were found at all the time points and across all three conditions tested (dialysis, dilution and equilibrium) (Figure 6g–i).

### 3.2. Scalability of the Microfluidic Method for Production of iLNPs (GenVoy-ILM™)

**Figure 7:**
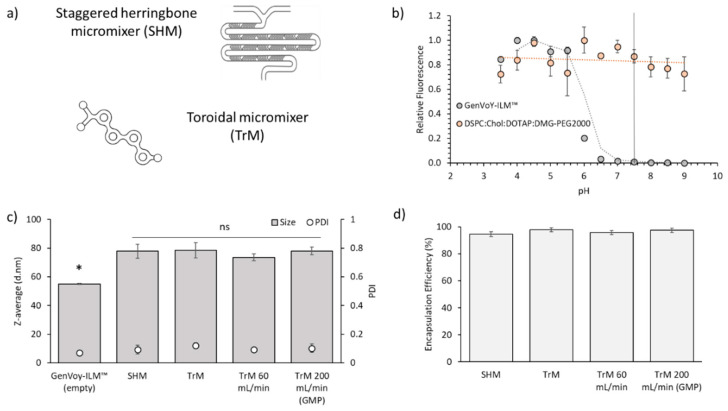
Scalable manufacture of iLNPs. (**a**) Schematic representation of the staggered herringbone micromixer (SHM) and toroidal mixer (TrM) used for the microfluidic production of LNPs. (**b**) Fluorescence titration curves of TNS with DSPC:Chol:DOTAP:DMG-PEG2000 cLNPs (orange circles) and GenVoy-ILM™ iLNPs (grey circles) at various pHs for experimentally arriving at surface pKa values. (**c**) Particle size (bars) and PDI (open circles) and (**d**) PolyA encapsulation efficiency of the LNPs (GenVoy-ILM™; a proprietary lipid mix comprising DSPC:Chol:ionisable lipid:stabiliser, loaded with PolyA at different production speeds (from 12 to 200 mL/min). Results represent the mean of at least 3 independent batches and the standard deviation is plotted as error bars. Significant differences are shown as * *p* < 0.05, and ‘ns’, when no statistical significance observed.

The data shown previously was generated using a SHM (Figure 7a). Hence, we have explored another microfluidic mixer with a TrM as described in a previous publication [14]. For this experiment, the GenVoy-ILM™ lipid mix composition commercially available from Precision NanoSystems was selected; this formulation contains an ionisable lipid different from MC3, as the main component. This ionisable lipid has an apparent pKa of approximately 6.0 (Figure 7b). The pKa is the pH at which 50% of the lipid molecules become positively charged. This can be measured using the TNS assay where this anionic molecule interacts with cationic particles and emits fluorescence. Blank and PolyA-loaded GenVoy-ILM™ iLNPs were then produced using the SHM and the TrM at 12, 60 and 200 mL/min TFR (GMP) (Figure 7c). The blank formulation displayed significantly (*p* < 0.05) smaller size (≈55 nm size) compared to its PolyA-loaded counterpart (≈78 nm). Both micromixers showed comparable sizes at all the speeds tested (Figure 7c), demonstrating the scalability of this microfluidic technology. In addition to this, high PolyA encapsulation efficiencies (EE ≥ 95%) were achieved (Figure 7d).

### 3.3. Encapsulation of Nucleic Acids into LNPs

**Figure 8:**
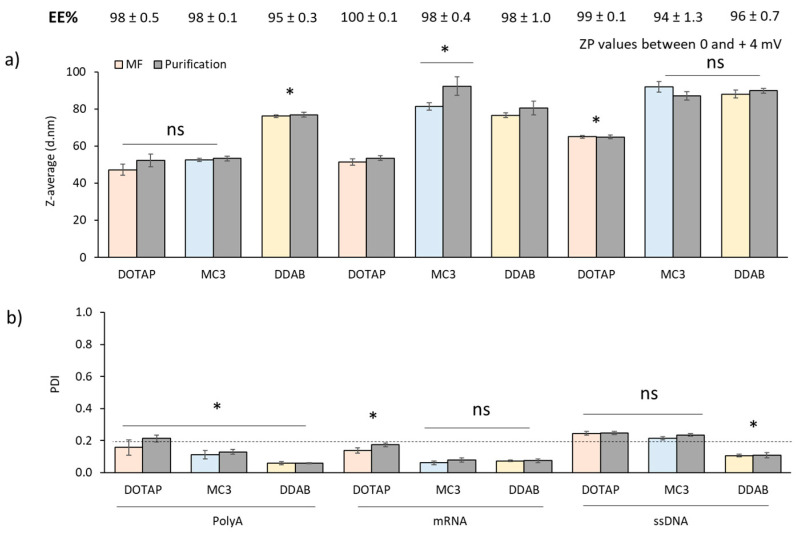
Loading of PolyA, ssDNA and mRNA on iLNPs (MC3) and cLNPs (DOTAP and DDAB) using citrate buffer (pH 6, 100 mM) at N/P 8 3:1 FRR and 20 mL/min TFR. (**a**) Particle size and (**b**) PDI of the DSPC:Chol:DOTAP:DMG-PEG2000 (pink), DSPC:Chol:MC3:DMG-PEG2000 (blue) and DSPC:Chol:DDAB:DMG-PEG2000 (yellow) before and after purification. Encapsulation efficiencies (EE%) for each formulation are shown as the percentage of the initial amount added (values). All formulations were prepared at 10:48:40:2 DSPC:Chol:cationic/ionisable:PEG lipid molar ratios. Results represent the mean of at least 3 independent batches and the standard deviation is plotted as error bars. Significant differences are shown as * *p* < 0.05, and ‘ns’, when no statistical significance observed.

**Figure 9:**
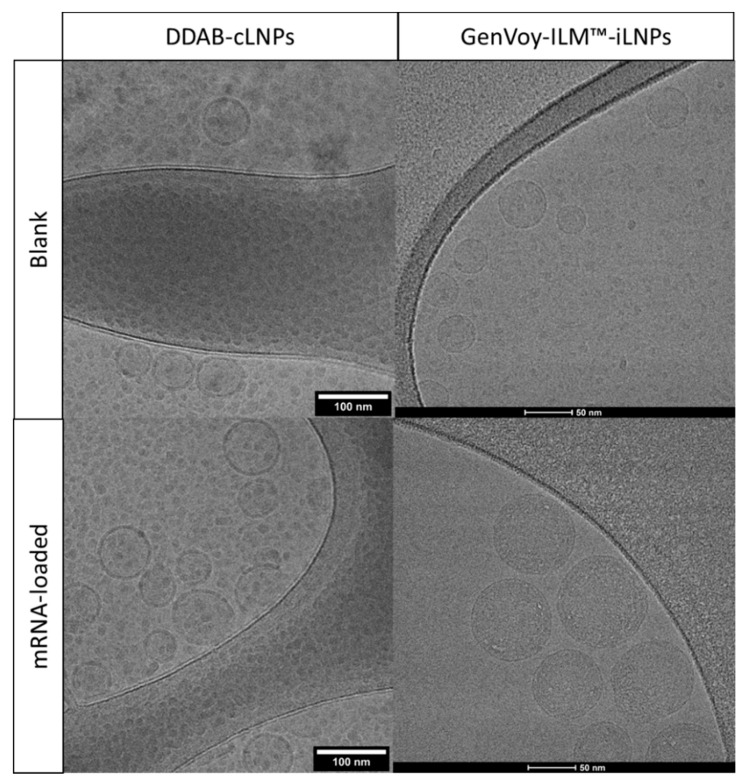
Cryo-TEM images of blank and mRNA-loaded DDAB-cLNPs (40,000× magnification, 200 kV) and GenVoy-ILM™ iLNPs (96,000× magnification, 300 kV).

After evaluation of the formulation and microfluidic operating parameters on the physicochemical characteristics of the empty LNPs, the next step was to determine the impact of the encapsulation of nucleic acids within these systems. PolyA, ssDNA and mRNA were selected for this study. Therefore, loaded LNPs were produced at 3:1 FRR and 20 mL/min TFR by adding the nucleic acid in the aqueous phase (citrate buffer pH 6 100 mM). All three nucleic acids were encapsulated at a fixed nitrogen to phosphate (N/P) mole ratio of 8. Figure 8 shows the particle size, PDI and zeta potential of the loaded LNPs before and after purification. PolyA-loaded LNPs showed the same particle sizes as their empty counterparts (≈47, 52 and 76 nm for DOTAP, MC3 and DDAB-LNPs respectively). However, when mRNA or ssDNA was encapsulated into these systems, the particle size increased (Figure 8). Particle sizes remained comparable after purification and the PDI across all formulations tested was below 0.25 (Figure 8a,b) and high EEs were achieved for all the LNP systems (>95%) (Figure 8). Cryo-TEM was used for the qualitative analysis of morphological features of the DDAB-cLNPs and GenVoy-ILM™ blank and mRNA-loaded formulations (Figure 9). Spherical sub-100 nm particle size ranges were observed with both DDAB cLNPs and GenVoy-ILM™ iLNPs. We observed that DDAB cLNPs had a very low electron dense aqueous-filled core containing spherical structure while the GenVoy-ILM™ iLNPs showing electron dense core containing spherical structure.

## 4. Discussions

Since the approval of Onpattro^®^ in 2018 by the Food and Drug Administration, LNPs containing ionisable lipids have been extensively investigated for the delivery of nucleic acids. Appropriate LNP designs usually differ depending on the nucleic acid payload and the administration route. For example, some studies have shown that the optimal pKa value for siRNA-iLNPs is approximately 6.4 [7], whereas for mRNA-iLNPs it is between 6.6–6.8 [17,18]. In general, optimal parameters consist of convenient particle size (usually below 100 nm to allow for sterile filtration and effective nucleic acid delivery), adequate composition, high encapsulation efficiencies (>90%), stability upon storage and a reproducible and scalable manufacturing method. Microfluidics has previously been shown to be an effective tool for the manufacture of LNPs [15,16,19,20], liposomes [21,22,23] and polymer-based nanoparticles [24,25,26]. However, the literature lacks reports evaluating the effects of the formulation and microfluidic operating parameters on the physicochemical characteristics of the LNPs. Within this study, we evaluated the effect of the total flow rate (TFR) and flow rate ratio (FRR), as well as the impact of the choice of structural lipid, PEG-lipid and aqueous buffer on the particle size, PDI and zeta potential of LNPs. Moreover, we encapsulated three types of nucleic acids within LNPs to assess their effect on the LNP physicochemical characteristics and on the encapsulation efficiency. Since microfluidics is based on the mixing of two liquid streams (lipids dissolved in organic solvent are mixed with an aqueous phase containing a nucleic acid at specific FRR and at selected TFR), we initially evaluated the effect of these operating parameters on three LNP systems consisting on DSPC, cholesterol, cationic (DOTAP or DDAB) or ionisable lipid (MC3) and DMG-PEG2000 at 10:48:40:2 molar ratio respectively. Lou et al. [13] produced a panel of cationic LNPs encapsulating a self-amplifying mRNA (N/P 8) using the same microfluidic technology used in this study. These cLNPs were compared to previously described MC3-based iLNPs [15]. All these LNPs were produced in methanol at 10:48:40:2 molar ratio at 3:1 FRR and 5 mL/min TFR. From this study, cLNPs based on DDAB and DOTAP showed the highest potency in vitro and induced comparable antibody responses to the benchmark iLNPs. Therefore, due to their good efficacy in vivo and the commercial availability to purchase these cationic/ionisable lipids, we selected these three LNPs (DDAB, DOTAP and MC3-based) for our microfluidic manufacture optimisation. We first optimized the production speed, which is an important parameter to consider, as this could restrict the manufacturing time. The majority of the literature reports no impact of this parameter on the manufacture of lipid-based delivery systems [14,22,27]. However, we have previously reported a reduction in the particle size of cationic liposomes based on DDAB:TDB when increasing the flow rate from 5 to 15 mL/min [21]. Here, we have shown that, at low TFR, the particles are significantly (*p* < 0.05) larger (Figure 2). Therefore, we hypothesize that the reduced mixing within the microfluidic micromixer at 5 mL/min results in a slow dilution of the solvent, promoting the formation of larger particles. On the other hand, the effect of the FRR on vesicle physicochemical characteristics has been extensively reported [14,22,23,28]. In general, increasing the FRR, and therefore, increasing polarity, creates a narrow organic solvent stream, which aids the formations of smaller particles due to reduced particle fusion [11]. In addition to this, here we observed destabilisation and stabilisation of LNPs when formulated at 1:1 FRR (Figure 3). It has been reported in the literature that the production of LNPs at 1:1 FRR using a T-shape mixer gave rise to metastable particles which required further dilution to be stabilised [29]. This could explain the results observed in Figure 3. DOTAP-cLNPs stabilised after the removal of solvent via dialysis; however, MC3-iLNPs became more unstable after dialysis. These results suggest that MC3 is more sensitive to organic solvent, and a faster removal or decrease of solvent content within the formulation (e.g., dilution) may improve the physicochemical characteristics of these iLNPs.

Next, we evaluated the effect of the individual lipid content on the size, PDI and zeta potential of the cLNP and iLNP systems (Figure 4). The aim of this experiment was to assess if minimal and large modifications on the molar lipid content could be detected by the physicochemical characteristics of the formed particles. Process analytical techniques such as AT-line sizers could be implemented in the manufacture process and therefore, this would allow for the quick detection of deviations in LNP composition if large production of LNPs is required [23]. LNPs generally require four components: a phospholipid, cholesterol, a permanent cationic or ionisable lipid and a PEG-lipid. Each of these components has a specific role within the formulation. There is limited information about the role of phospholipids (commonly DSPC) and cholesterol in the activity of LNPs [30]. However, it has been reported that both DSPC and cholesterol, promote stability, contributing to the structural integrity of the formulation [31]. In addition to this, DSPC increases membrane rigidity and decreases membrane permeability due to its high transition temperature (55 °C). Some studies have reported that cholesterol-free or cholesterol-deficient LNPs might have possible destabilisation effects which might result in decreased LNP potency [32,33]. These lipids aid in the encapsulation of the nucleic acids. PEG-lipid plays several roles. It is located on the surface of the LNPs and it dictates the particle size of the LNPs during manufacture [16]. Production of LNPs in acidic pH without PEG-lipid would promote particle fusion [9]. In addition to this, they increase product shelf-life and in vivo circulation lifetime [34]. Finally, the role of the ionisable or cationic lipid is to both promote electrostatic interaction with the negatively charged nucleic acids and promote intracellular delivery. Moreover, ionisable lipids facilitate endosomal escape through fusion with the endosome membrane as a result of the cationic charge under endosomal pH [35].

We further explored the effect of replacing the phospholipid or the PEG-lipid into the particle size of the LNPs. No impact on the physicochemical characteristics of the DOTAP-cLNPs was observed (Figure 5). It has been reported elsewhere that the type of phospholipid selected for formulation within the LNPs influences the LNP potency [18]. Regarding the choice of PEG-lipid, short hydrocarbon chains (C14 -DMG-PEG2000) that diffuse out of the LNPs enhance intracellular delivery [34,36,37]. However, C18 chains (DSPE-PEG2000), do not dissociate as easily, contributing to an extended circulation lifetime [38]. As well as lipid content, another important parameter to consider is the aqueous buffer. Citrate buffer at a low pH is commonly used for the production of RNA LNPs since it reduces the hydrolysis of RNA due to the chelating effect of sodium citrate. However, for experimental purposes, we sought to study the effect that the well-known TRIS buffer (commonly prepared at physiological, pH 7.4) had on the LNP production by microfluidics. The non-ionisable DOTAP-cLNPs were selected for this purpose. TRIS demonstrated an impact on the zeta potential of the cLNPs, displaying positive zeta potentials compared to the neutral charge observed on cLNPs produced using citrate buffer. It is hypothesised that the reactive amine group within the TRIS structure becomes protonated, resulting in cationic zeta potentials. Moreover, the effect that buffer type and concentration have on liposomal systems produced using microfluidics has been previously explained by Lou et al. [39]. Lou et al. demonstrated that DSPC:Chol:DOTAP liposomes were larger (approx. 100 nm) when prepared using citrate buffer pH 6 compared to TRIS buffer pH 7.4 (approx. 60 nm) even at the same buffer molarity [39]. Particle size was dependent on buffer type, concentration and cationic lipid content. The higher the cationic lipid content or the buffer concentration, the larger the particle size [38]. It was hypothesised that the presence of electrolytes may decrease lipid head repulsion, affecting the packing of the lipids and thus producing large cationic liposomes. Furthermore, we observed that using TRIS buffer for the preparation of DOTAP-based LNPs at high flow rates (20 mL/min) produced small and unstable LNPs (Figure 6). We hypothesise that a combination of both high production speeds and buffer choice contribute to the formation of very small particles that need more time to form, and thus, after equilibrium or dilution, these particles reach their stability. However, we have also demonstrated that particle stability can be achieved by reducing the flow rate (TFR) or by encapsulating nucleic acids (Figure 6). The encapsulation of PolyA contributed to the stabilisation of the particles by means of the electrostatic interactions between the positively charged LNPs and the negatively charged nucleic acid.

Encapsulation of a nucleic acids with different molecular weights resulted in particle sizes below 100 nm (Figure 8), which have been reported in the literature to be more potent than larger particle sizes, with optimum LNP potency displayed at approximately 80 nm [37,40]. Moreover, there was a consistent range of particle sizes, as indicated by DLS and Cryo-TEM. Lou et al. produced self-amplifying mRNA-LNPs with the same lipid composition as the ones tested in this study, with particle sizes between 80–100 nm, a narrow size distribution and high encapsulation efficiencies (>95%) [13]. Blakney et al. also prepared self-amplifying mRNA-loaded LNPs based on the cationic lipids DOTAP and DDAB or on the ionisable lipid C12–200 [41]. These LNPs were prepared using a microfluidic platform different from the one presented here, and no significant differences were observed on their physicochemical characteristics (100–200 nm, PDI ~0.2) [41]. Morphological characterisation of blank and mRNA-loaded DDAB-cLNPs and GenVoy-ILM™-iLNPs (Figure 9) showed comparable morphology to what it has previously been reported in literature and particle sizes were in agreement with the DLS measurements (e.g., [13,42]). Blank and mRNA-loaded iLNPs revealed an electron dense core, suggesting that the ionisable lipid contributes to this appearance when not complexed to nucleic acids [43]. On the other hand, blank DDAB-cLNPs showed a less dense interior [13].

The use of SHM allows for rapid and reproducible manufacture at bench to clinic scale. GMP production using a SHM would result in an expensive task, due to the need of parallelization of the micromixers. However, the use of a TrM represents an advantage since it allows higher throughputs, favouring the progression from bench (mL) to GMP-scale (L) [14,44]. We have previously demonstrated the ability of the TrM to produce protein-loaded liposomes on a large scale (L) and high speeds (200 mL/min) [14]. Here, we have demonstrated the scalability of microfluidics for the manufacture of nucleic acid-loaded iLNPs at high production speeds required for large-scale GMP-manufacturing conditions (Figure 7). This is a hot topic nowadays, since due to the SARS-CoV-19 pandemic, there is a high demand for scalable techniques for mass vaccination, and there are currently a number of vaccines in clinical trials based on RNA-LNP systems.

## 5. Conclusions

Here, we have shown a comprehensive study of the effect of the formulation components and the microfluidic process parameters on the physicochemical characteristics of LNPs. The cationic (DDAB and DOTAP) and ionisable (MC3) LNPs tested have shown high sensitivity to the microfluidic operating parameter flow rate ratio and the total flow rate. Swapping structural lipids or PEG-lipids resulted in similar trends being interchangeable. Buffer choice and molar lipid content are important considerations for LNP manufacture, as these influenced LNP size and stability. In addition to this, microfluidics allows for a wide range of nucleic acids (e.g., PolyA, ssDNA, mRNA) to be accommodated. This method of optimisation and formulation screening could be used as a guide for the preparation of LNPs for gene delivery.

## Data Access

All data underpinning this publication is openly available from the University of Strathclyde KnowledgeBase at https://doi.org/10.15129/32db8191-3d6f-4d44-ade0-72c285f82a99.

## References

[1] Dammes N., Peer D. (2020). Paving the road for RNA therapeutics. Trends Pharmacol. Sci..

[2] Park J., Park J., Pei Y., Xu J., Yeo Y. (2016). Pharmacokinetics and biodistribution of recently-developed siRNA nanomedicines. Adv. Drug Deliv. Rev..

[3] Kaczmarek J.C., Kowalski P.S., Anderson D.G. (2017). Advances in the delivery of RNA therapeutics: From concept to clinical reality. Genome Med..

[4] U.S. Food and Drug Administration Drug Approval Package: Onpattro (Patisiran). https://www.accessdata.fda.gov/drugsatfda_docs/nda/2018/210922Orig1s000TOC.cfm.

[5] U.S. Food and Drug Administration Drug Approval Package: GIVLAARI (Givosiran) Injection. https://www.accessdata.fda.gov/drugsatfda_docs/nda/2019/212194Orig1s000TOC.cfm.

[6] Semple S.C., Klimuk S.K., Harasym T.O., Dos Santos N., Ansell S.M., Wong K.F., Maurer N., Stark H., Cullis P.R., Hope M.J. (2001). Efficient encapsulation of antisense oligonucleotides in lipid vesicles using ionizable aminolipids: Formation of novel small multilamellar vesicle structures. Biochim. Biophys. Acta (BBA) Biomembr..

[7] Jayaraman M., Ansell S.M., Mui B.L., Tam Y.K., Chen J., Du X., Butler D., Eltepu L., Matsuda S., Narayanannair J.K. (2012). Maximizing the Potency of siRNA Lipid Nanoparticles for Hepatic Gene Silencing In Vivo. Angew. Chem. Int. Ed..

[8] Akinc A., Maier M.A., Manoharan M., Fitzgerald K., Jayaraman M., Barros S., Ansell S., Du X., Hope M.J., Madden T.D. (2019). The Onpattro story and the clinical translation of nanomedicines containing nucleic acid-based drugs. Nat. Nanotechnol..

[9] Samaridou E., Heyes J., Lutwyche P. (2020). Lipid nanoparticles for nucleic acid delivery: Current perspectives. Adv. Drug Deliv. Rev..

[10] MacLachlan I. (2007). Liposomal formulations for nucleic acid delivery. Antisense Drug Technol. Princ. Strat. Appl..

[11] Jahn A., Vreeland W.N., Gaitan M., Locascio L.E. (2004). Controlled Vesicle Self-Assembly in Microfluidic Channels with Hydrodynamic Focusing. J. Am. Chem. Soc..

[12] Wagner A., Vorauer-Uhl K., Kreismayr G., Katinger H. (2002). The crossflow injection technique: An improvement of the ethanol injection method. J. Liposome Res..

[13] Lou G., Anderluzzi G., Schmidt S.T., Woods S., Gallorini S., Brazzoli M., Giusti F., Ferlenghi I., Johnson R.N., Roberts C.W. (2020). Delivery of self-amplifying mRNA vaccines by cationic lipid nanoparticles: The impact of cationic lipid selection. J. Controlled Rel..

[14] Webb C., Forbes N., Roces C.B., Anderluzzi G., Lou G., Abraham S., Ingalls L., Marshall K., Leaver T.J., Watts J.A. (2020). Using microfluidics for scalable manufacturing of nanomedicines from bench to GMP: A case study using protein-loaded liposomes. Int. J. Pharm..

[15] Geall A.J., Verma A., Otten G.R., Shaw C.A., Hekele A., Banerjee K., Cu Y., Beard C.W., Brito L.A., Krucker T. (2012). Nonviral delivery of self-amplifying RNA vaccines. Proc. Natl. Acad. Sci. USA.

[16] Belliveau N.M., Huft J., Lin P.J.C., Chen S., Leung A.K.K., Leaver T.J., Wild A.W., Lee J.B., Taylor R.J., Tam Y.K. (2012). Microfluidic Synthesis of Highly Potent Limit-size Lipid Nanoparticles for In Vivo Delivery of siRNA. Mol. Ther. Nucleic Acids.

[17] Hassett K.J., Benenato K.E., Jacquinet E., Lee A., Woods A., Yuzhakov O., Himansu S., Deterling J., Geilich B.M., Ketova T. (2019). Optimization of Lipid Nanoparticles for Intramuscular Administration of mRNA Vaccines. Mol. Ther. Nucleic Acids.

[18] Kauffman K.J., Dorkin J.R., Yang J.H., Heartlein M.W., DeRosa F., Mir F.F., Fenton O.S., Anderson D.G. (2015). Optimization of Lipid Nanoparticle Formulations for mRNA Delivery in Vivo with Fractional Factorial and Definitive Screening Designs. Nano Lett..

[19] Leung A.K., Tam Y.Y., Chen S., Hafez I.M., Cullis P.R. (2015). Microfluidic Mixing: A General Method for Encapsulating Macromolecules in Lipid Nanoparticle Systems. J. Phys. Chem. B.

[20] Maeki M., Saito T., Sato Y., Yasui T., Kaji N., Ishida A., Tani H., Baba Y., Harashima H., Tokeshi M. (2015). A strategy for synthesis of lipid nanoparticles using microfluidic devices with a mixer structure. RSC Adv..

[21] Roces C.B., Khadke S., Christensen D., Perrie Y. (2019). Scale-Independent Microfluidic Production of Cationic Liposomal Adjuvants and Development of Enhanced Lymphatic Targeting Strategies. Mol. Pharm..

[22] Forbes N., Hussain M.T., Briuglia M.L., Edwards D.P., Horst J.H.t., Szita N., Perrie Y. (2019). Rapid and scale-independent microfluidic manufacture of liposomes entrapping protein incorporating in-line purification and at-line size monitoring. Int. J. Pharm..

[23] Roces C.B., Port E.C., Daskalakis N.N., Watts J.A., Aylott J.W., Halbert G.W., Perrie Y. (2020). Rapid scale-up and production of active-loaded PEGylated liposomes. Int. J. Pharm..

[24] Roces C.B., Christensen D., Perrie Y. (2020). Translating the fabrication of protein-loaded poly (lactic-co-glycolic acid) nanoparticles from bench to scale-independent production using microfluidics. Drug Deliv. Transl. Res..

[25] Roces C.B., Hussain M.T., Schmidt S.T., Christensen D., Perrie Y. (2019). Investigating Prime-Pull Vaccination through a Combination of Parenteral Vaccination and Intranasal Boosting. Vaccines.

[26] Anderluzzi G., Lou G., Gallorini S., Brazzoli M., Johnson R., O’Hagan D.T., Baudner B.C., Perrie Y. (2020). Investigating the Impact of Delivery System Design on the Efficacy of Self-Amplifying RNA Vaccines. Vaccines.

[27] Zizzari A., Bianco M., Carbone L., Perrone E., Amato F., Maruccio G., Rendina F., Arima V. (2017). Continuous-flow production of injectable liposomes via a microfluidic approach. Materials.

[28] Jahn A., Stavis S.M., Hong J.S., Vreeland W.N., DeVoe D.L., Gaitan M. (2010). Microfluidic mixing and the formation of nanoscale lipid vesicles. ACS Nano.

[29] Jeffs L.B., Palmer L.R., Ambegia E.G., Giesbrecht C., Ewanick S., MacLachlan I. (2005). A Scalable, Extrusion-Free Method for Efficient Liposomal Encapsulation of Plasmid DNA. Pharm. Res..

[30] Kulkarni J.A., Myhre J.L., Chen S., Tam Y.Y.C., Danescu A., Richman J.M., Cullis P.R. (2017). Design of lipid nanoparticles for in vitro and in vivo delivery of plasmid DNA. Nanomed. Nanotechnol. Biol. Med..

[31] Cheng X., Lee R.J. (2016). The role of helper lipids in lipid nanoparticles (LNPs) designed for oligonucleotide delivery. Adv. Drug Deliv. Rev..

[32] Rodrigueza W.V., Wheeler J.J., Klimuk S.K., Kitson C.N., Hope M.J. (1995). Transbilayer Movement and Net Flux of Cholesterol and Cholesterol Sulfate between Liposomal Membranes. Biochemistry.

[33] Sato Y., Okabe N., Note Y., Hashiba K., Maeki M., Tokeshi M., Harashima H. (2020). Hydrophobic scaffolds of pH-sensitive cationic lipids contribute to miscibility with phospholipids and improve the efficiency of delivering short interfering RNA by small-sized lipid nanoparticles. Acta Biomater..

[34] Holland J.W., Hui C., Cullis P.R., Madden T.D. (1996). Poly(ethylene glycol)−Lipid Conjugates Regulate the Calcium-Induced Fusion of Liposomes Composed of Phosphatidylethanolamine and Phosphatidylserine. Biochemistry.

[35] Guo X., Wang H., Li Y., Leng X., Huang W., Ma Y., Xu T., Qi X. (2019). Transfection reagent Lipofectamine triggers type I interferon signaling activation in macrophages. Immunol. Cell Biol..

[36] Judge A., McClintock K., Phelps J.R., MacLachlan I. (2006). Hypersensitivity and Loss of Disease Site Targeting Caused by Antibody Responses to PEGylated Liposomes. Mol. Ther..

[37] Chen S., Tam Y.Y.C., Lin P.J.C., Sung M.M.H., Tam Y.K., Cullis P.R. (2016). Influence of particle size on the in vivo potency of lipid nanoparticle formulations of siRNA. J. Controlled Rel..

[38] Mui B.L., Tam Y.K., Jayaraman M., Ansell S.M., Du X., Tam Y.Y.C., Lin P.J.C., Chen S., Narayanannair J.K., Rajeev K.G. (2013). Influence of Polyethylene Glycol Lipid Desorption Rates on Pharmacokinetics and Pharmacodynamics of siRNA Lipid Nanoparticles. Mol. Ther. Nucleic Acids.

[39] Lou G., Anderluzzi G., Woods S., Roberts C.W., Perrie Y. (2019). A novel microfluidic-based approach to formulate size-tuneable large unilamellar cationic liposomes: Formulation, cellular uptake and biodistribution investigations. Eur. J. Pharm. Biopharm..

[40] Sato Y., Hatakeyama H., Hyodo M., Harashima H. (2016). Relationship Between the Physicochemical Properties of Lipid Nanoparticles and the Quality of siRNA Delivery to Liver Cells. Mol. Ther..

[41] Blakney A.K., McKay P.F., Yus B.I., Aldon Y., Shattock R.J. (2019). Inside out: Optimization of lipid nanoparticle formulations for exterior complexation and in vivo delivery of saRNA. Gene Ther..

[42] Kulkarni J.A., Witzigmann D., Leung J., Tam Y.Y.C., Cullis P.R. (2019). On the role of helper lipids in lipid nanoparticle formulations of siRNA. Nanoscale.

[43] Leung A.K.K., Hafez I.M., Baoukina S., Belliveau N.M., Zhigaltsev I.V., Afshinmanesh E., Tieleman D.P., Hansen C.L., Hope M.J., Cullis P.R. (2012). Lipid Nanoparticles Containing siRNA Synthesized by Microfluidic Mixing Exhibit an Electron-Dense Nanostructured Core. J. Phys. Chem. C.

[44] Lee C.-Y., Wang W.-T., Liu C.-C., Fu L.-M. (2016). Passive mixers in microfluidic systems: A review. Chem. Eng. J..

